# Acute exercise does not decrease liver fat in men with overweight or NAFLD

**DOI:** 10.1038/srep09709

**Published:** 2015-04-13

**Authors:** L. Bilet, B. Brouwers, P. A. van Ewijk, M. K. C. Hesselink, M. E. Kooi, P. Schrauwen, V. B. Schrauwen-Hinderling

**Affiliations:** 1 NUTRIM, School of Nutrition and Translational Research in Metabolism, Maastricht University Medical Center, Maastricht, The Netherlands; 2 Department of Human Biology, Maastricht University Medical Center, Maastricht, The Netherlands; 3 Department of Radiology, Maastricht University Medical Center, Maastricht, The Netherlands; 4 Department of Human Movement Sciences, Maastricht University Medical Center, Maastricht, The Netherlands; 5 CARIM, Cardiovascular Research Institute Maastricht, Maastricht University Medical Center, Maastricht, The Netherlands

## Abstract

Elevated hepatic lipid content (IntraHepatic Lipid, IHL) increases the risk of metabolic complications. Although prolonged exercise training lowers IHL, it is unknown if acute exercise has the same effect. Furthermore, hepatic ATP content may be related to insulin resistance and IHL. We aimed to investigate if acute exercise leads to changes in IHL and whether this is accompanied by changes in hepatic ATP. Twenty-one men (age 54.8 ± 7.2 years, BMI 29.7 ± 2.2 kg/m^2^) performed a 2 h cycling protocol, once while staying fasted and once while ingesting glucose. IHL was determined at baseline, 30 min post-exercise and 4 h post-exercise. Additionally ATP/Total P ratio was measured at baseline and 4 h post-exercise. Compared with baseline values we did not observe any statistically significant changes in IHL within 30 min post-exercise in neither the fasted nor the glucose-supplemented condition. However, IHL was elevated 4 h post-exercise compared with baseline in the fasted condition (from 8.3 ± 1.8 to 8.7 ± 1.8%, p = 0.010), an effect that was blunted by glucose supplementation (from 8.3 ± 1.9 to 8.3 ± 1.9%, p = 0.789). Acute exercise does not decrease liver fat in overweight middle-aged men. Moreover, IHL increased 4 h post-exercise in the fasted condition, an increase that was absent in the glucose-supplemented condition. These data suggest that a single bout of exercise may not be able to lower IHL.

Regular exercise has beneficial effects on metabolic risk factors associated with type 2 diabetes^1^,^2^. Recent studies have suggested that prolonged exercise training reduces liver fat content and may thereby contribute to the beneficial effects of exercise on metabolic risk^3^,^4^,^5^,^6^. Results from exercise training studies in animals revealed that the effect of exercise on hepatic lipid content (IntraHepatic Lipid, IHL) strongly depends on the diet and that exercise is more effective in reducing IHL under conditions that favor liver fat accretion, such as when animals are fed a high-fat diet^7^. Interestingly, human data also revealed that exercise training appears to be more potent in reducing IHL in subjects with increased baseline IHL. Thus, the exercise-mediated reduction in IHL is more pronounced in subjects with Non-alcoholic fatty liver disease (NAFLD), type 2 diabetes, or in elderly^8^ than in healthy normal weight and young subjects. Furthermore, like with exercise training, acute bouts of exercise also improve insulin sensitivity^9^,^10^,^11^. The acute effects of exercise on IHL, however, have not yet been intensively studied. As yet, only three studies have examined the effect of acute exercise on IHL. However, all three of these studies were performed in lean young healthy volunteers in which liver fat is usually rather low^12^,^13^,^14^, which would complicate detecting exercise-mediated reductions in IHL. To circumvent this, one study^12^ let the male subjects consume a high-fat diet or a mixed diet for 67 h before exercise. Subsequently IHL was measured (with ^1^H-MRS) before and after 90 minutes of moderate intensity cycling. No statistically significant decrease was found directly after exercise, irrespective of the dietary condition. Hence it was concluded that acute exercise does not lower liver fat in young healthy men. In the other two studies, one study including male subjects with an average age of 28.9 years old^13^ and one study including male and female subjects with a slightly older age of 37.6 years old^14^, subjects also consumed a high-fat diet 3 days prior to the exercise trial. Here IHL (measured with ^1^H-MRS) was increased rather than decreases after a 2 hours exercise bout (50–60% of VO2 max), and it was speculated that the increase in IHL upon exercise was due to increased free fatty acid (FFA) availability during exercise. A clinically more relevant question, however, would be if acute exercise lowers liver fat in a middle-aged overweight subject population who are prone to the development of fatty liver or may already have elevated IHL (NAFLD), but such information is presently lacking.

As mentioned above, a potentially biasing complication when measuring IHL post-exercise is the increase in plasma FFA that goes along with exercise in the fasted state (originating from exercise-mediated increase in adipose tissue lipolysis)^15^,^16^. Plasma FFA is an important source for hepatic triglyceride^17^, and therefore high levels of plasma FFA during exercise might mask a potential exercise-lowering effect. To circumvent this bias, we here investigated the effect of acute exercise on IHL in middle-aged overweight sedentary subjects with a wide range of liver fat content under conditions with high and low plasma FFA concentrations.

Next to IHL, hepatic ATP concentrations, a measure for liver energy status, have also been suggested to be related to insulin resistance and hepatic lipid accumulation^18^. Therefore, we also studied if changes in IHL are accompanied by changes in hepatic ATP concentration. To this end, we employed ^1^H-MRS to determine IHL before and after exercise performed with and without glucose ingestion to suppress plasma FFA levels, as well as 4 h post exercise. ^31^P-MRS was employed before exercise and 4 h post exercise to determine hepatic ATP concentrations.

## Results

### Basic characteristics

**Figure 1:**
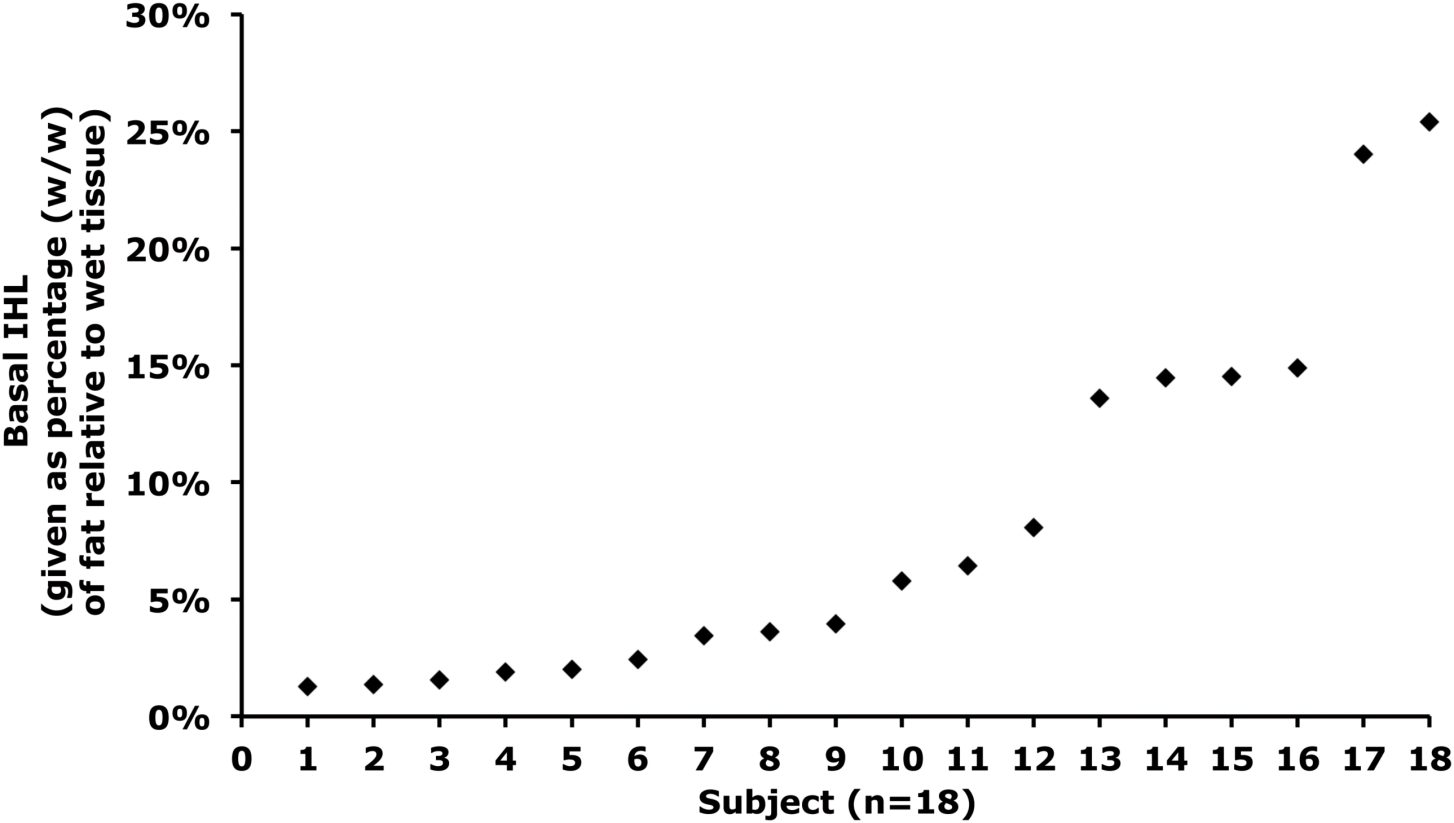
Hepatic lipid content at baseline for each individual subject given as percentage (w/w) of fat relative to wet tissue, n = 18.

Twenty-one middle-aged overweight men participated in this study. The subject characteristics of the entire group are shown in Table 1. The subjects had a wide variety of liver fat content (see figure 1 for basal liver fat content per subject). Of these subjects, eleven subjects met the clinical criteria for NAFLD (>5.6% liver fat)^19^, while in ten subjects hepatic fat content was within the normal physiological range (<5.6% liver fat). There were no significant differences in body weight, BMI, whole body fat percentage and fasting plasma glucose levels between subjects that would qualify as NAFLD and subjects with a normal liver fat content, but the NAFLD subjects were somewhat younger than the subjects with normal liver fat content (51.7 ± 5.4 years vs 58.2 ± 7.7 years, p = 0.036) and had a higher diastolic blood pressure (p = 0.011). As expected, clinical parameters that associate with fatty liver, such as plasma levels of TG and the liver enzymes gamma-GT, ASAT and ALAT were all significantly higher in subjects with a high (>5.6%) liver fat content compared to the subjects with normal liver fat content (p < 0.05). However, despite these clinical differences, liver fat content responded similarly in subjects with low and high liver fat content and hence all subjects are treated as one group in the data presented below.

### Energy expenditure and substrate oxidation

**Figure 2:**
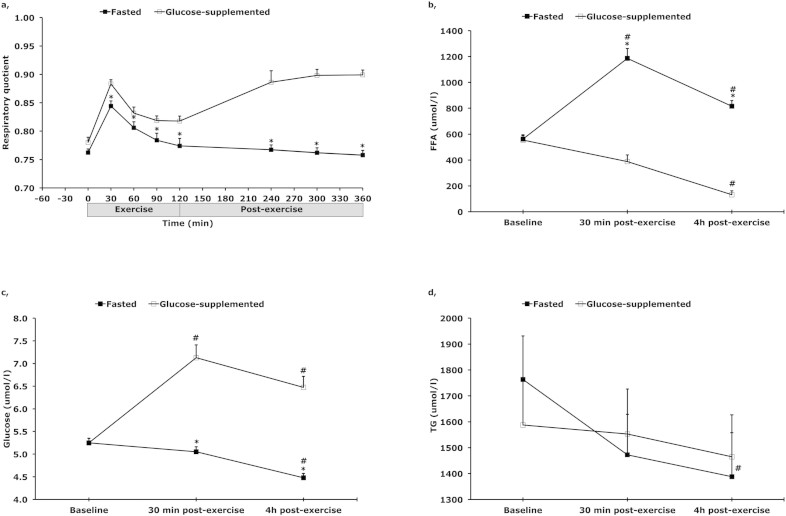
(a) Respiratory quotient during and after 2 h of cycling at 50% of maximal power output (Wmax) (n = 17) and plasma concentrations of (b) free fatty acids (FFA), (c) glucose, (d) and triglycerides (TG), with (open square) and without (filled square) glucose supplementation. * p < 0.05 compared with glucose-supplemented condition. # p < 0.05 compared with baseline (t = −60). Data are mean ± SE.

No significant differences in energy expenditure between the glucose-fed and the fasted state were found at baseline, during exercise or post exercise (see table 2). During exercise, there was a significant time (p < 0.001) and treatment (p < 0.001) effect, without a time*treatment interaction (p < 0.132) effect for respiratory quotient (RQ). RQ was significantly higher at every time point, except for baseline, in the glucose-fed state compared with the fasted state (p < 0.01) (see figure 2a), reflecting a higher carbohydrate oxidation in the glucose-fed state compared with the fasted state (p < 0.01) and a higher fat oxidation in the fasted state compared with the glucose-fed state (see table 2 for whole body glucose- and fat oxidation). RQ dropped between t = 30 and t = 120 min (p < 0.001) in both conditions. In the post-exercise period, a significant treatment effect (p < 0.001) was observed, with RQ remaining significantly higher in the glucose-fed state compared with the fasted state at every time point (p < 0.001). Data presented above is acquired from 17 subjects, data from four subjects had to be excluded from the analysis due to missing or poor quality of data.

### Plasma concentrations

There was a significant time (p < 0.0001), treatment (p < 0.0001) and time*treatment interaction (p < 0.0001) effect for plasma FFA concentrations. Plasma FFA concentrations increased with time during exercise and recovery from exercise in the fasted state, with a significant higher plasma FFA concentration at the end of exercise (t = 120) and 4 hours post-exercise (t = 360) compared with before exercise (t = −60) (p < 0.0001), whereas plasma FFA concentrations decreased over time in the glucose-fed state (p = 0.002). Furthermore, plasma FFA concentrations were substantially higher at the end of 2 hours of exercise and 4 hours post-exercise in the fasted state (p < 0.0001) compared with the glucose-fed state (Fig. 2b).

Plasma glucose concentrations showed a significant time (p < 0.0001), treatment (p < 0.0001) and time*treatment interaction (p < 0.0001) effect. Thus, plasma glucose concentrations increased with time during exercise and recovery from exercise in the glucose-supplemented state, with a significant higher plasma glucose concentration at the end of exercise (t = 120) and 4 hours post-exercise (t = 360) compared with before exercise (t = −60) (p = 0.0001), whereas plasma glucose concentrations decreased over time in the fasted state (p = 0.0001). Moreover, plasma glucose concentrations were higher at the end of 2 hours of exercise and 4 hours post-exercise in the glucose-supplemented state (p < 0.0001) compared with the fasted state (Fig. 2c).

Plasma triglyceride concentrations showed a significant time (p < 0.0001) and time*treatment interaction (p < 0.0001) effect in the fasted condition (Fig. 2d). Plasma triglyceride concentrations decreased with time from baseline (t = −60) to post-recovery (t = 360) in the fasted state, whereas it did not change when glucose supplementation was given.

### Hepatic lipid content

**Figure 3:**
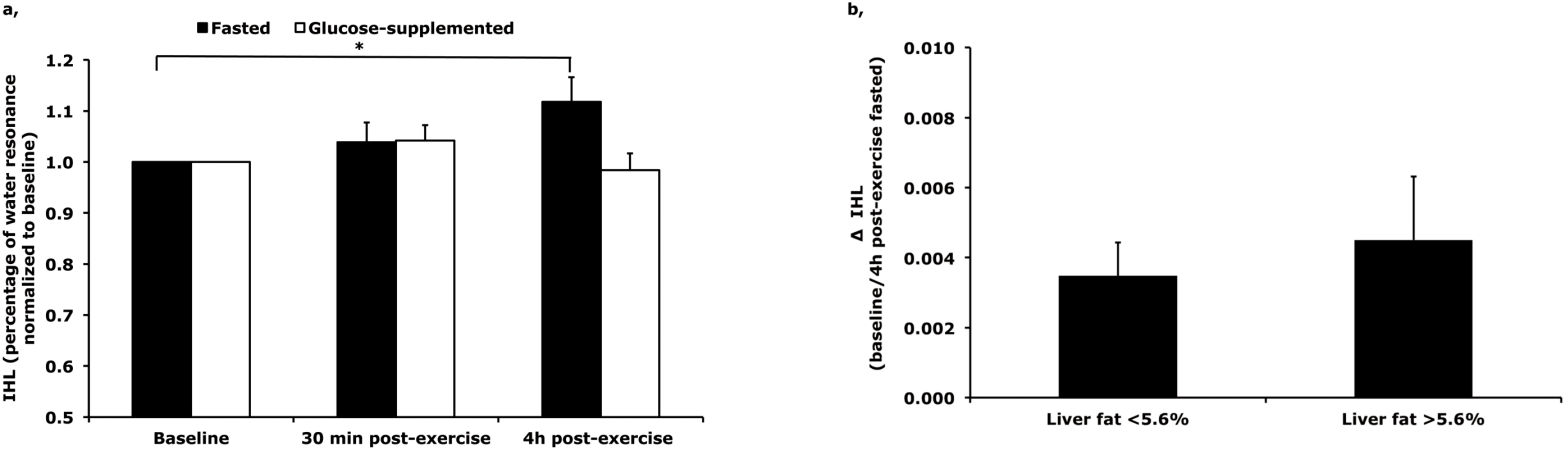
(a) Hepatic lipid content within 30 min after cessation of exercise (30 min post-exercise) and 4 h post-exercise, (because the subjects had a wide variety of liver fat content data was normalized to baseline values), n = 18, and (b) delta (Δ) IHL between baseline and 4 h post-exercise in the fasted condition in subjects with low (<5.6%) and high (>5.6%) liver fat content * p < 0.05. Data are mean ± SE.

Hepatic lipid content was investigated, by the means of ^1^H-MRS, before, within 30 min after cessation of exercise and 4 h post-exercise. IHL did not change significantly with acute exercise neither in the fasted nor in the glucose-supplemented condition, with IHL after cessation of exercise (t = 120) being 8.3 ± 1.9% of the water resonance in the fasted condition (p = 0.154) and 8.4 ± 1.8% in the glucose-supplemented condition (p = 0.181). However, IHL was increased 4 h post-exercise in the fasted condition compared with before exercise (8.3 ± 1.8% of the water resonance before exercise to 8.7 ± 1.8% of the water resonance 4 h post-exercise, (p = 0.010)); this increase in IHL post-exercise was absent in the glucose-supplemented condition (from 8.3 ± 1.9% to 8.3 ± 1.9% of the water resonance, (p = 0.789) (Fig. 3a).

When dividing subjects with low (<5.6%) and high (>5.6%) liver fat content, similar effects are found, with no significant changes in IHL within 30 min after cessation of exercise in both conditions and slightly increased 4 h post-exercise in the fasted condition. Furthermore, the increase in IHL 4 h post-exercise was similar for both groups (p = 0.630) (see delta increase in figure 3b).

### Hepatic ATP/Total P ratio

**Figure 4:**
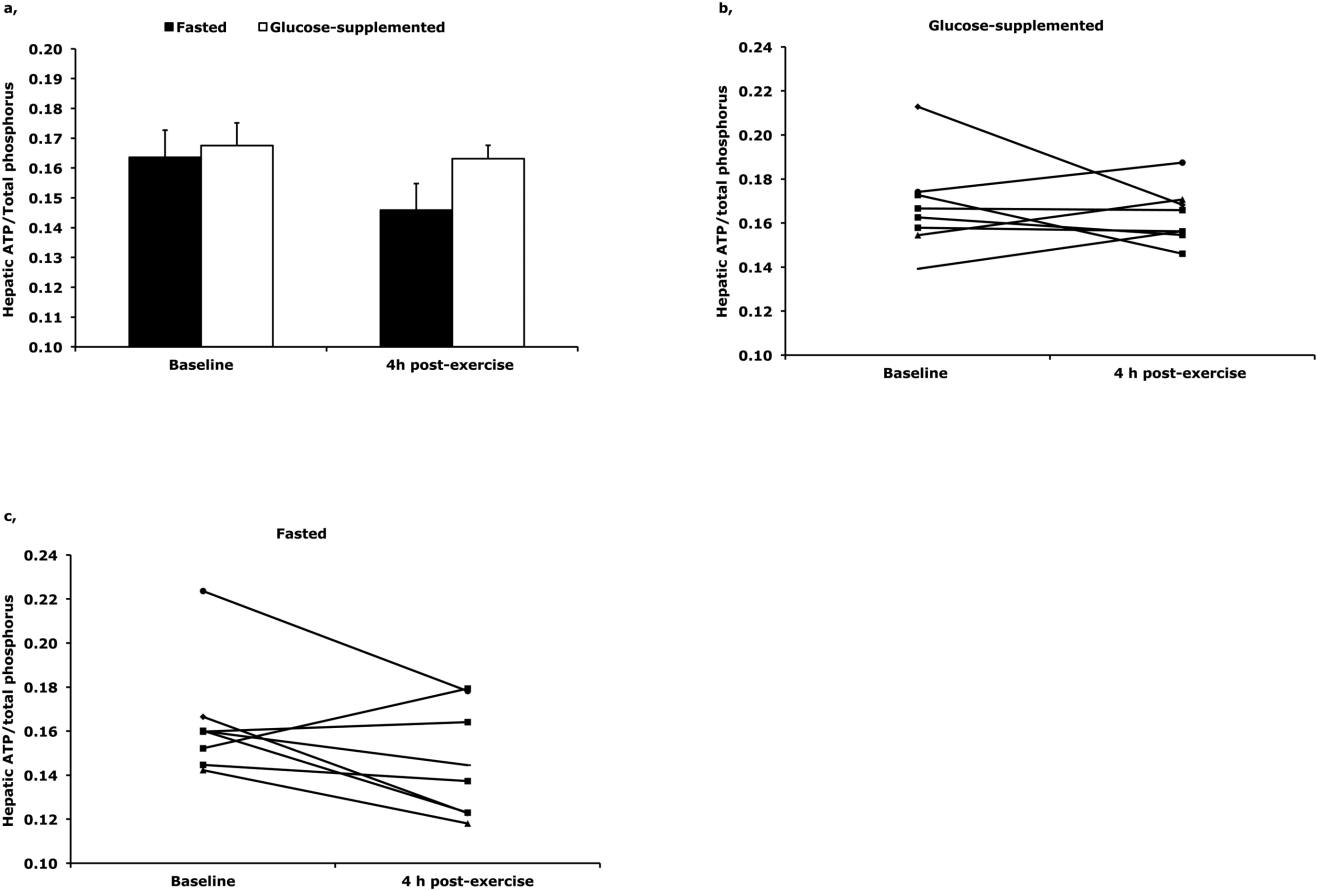
Hepatic ATP/Total P ratio (the relative amount of total phosphorous in the liver) at baseline and 4 h post-exercise as (a) group average and individual data with (b) and without (c) glucose supplementation. In the fasted condition (without glucose-supplementation) it was a trend to a lower hepatic ATP/Total P ratio 4 h post-exercise compared with baseline.

ATP/Total P ratio was not statistically significant different from baseline 4 h post-exercise in neither the fasted (p = 0.086) nor the glucose-supplemented (p = 0.582) condition (Fig. 4a). However, although not statistically significant, we observed a tendency to a decrease in ATP/Total P ratio in the fasted condition. In six out of eight subjects ATP/Total P ratio decreased with 16.9 ± 3.3% four hours post-exercise, whereas we did not observe a decrease in ATP/Total P ratio when glucose supplementation was given (See individual data in figure 4b and c). No correlation was found between IHL and hepatic ATP content in this subgroup of eight subjects.

## Discussion

Recent evidence suggests that prolonged exercise training might have a lowering effect on IHL^3^,^4^,^5^,^6^. Here, we examined the effect of an acute bout of exercise on the IHL in middle-aged overweight men with a wide range of liver fat content and we did not observe a decreased IHL in this population. Moreover, in the recovery phase after exercise, IHL was increased at 4 h post-exercise in the fasted condition; this increase in IHL was absent when subjects consumed glucose during and after exercise. During and after exercise, circulatory FFA levels are profoundly increased, which may deliver FFA to the liver with increased IHL as a consequence. Of note, glucose supplementation blunts the exercise-induced increase in plasma FFA levels. Therefore, the results of the present study indicate that in spite of strongly stimulated whole body fat oxidation during exercise, an acute bout of exercise is not enough to decrease IHL in this population.

There is an increasing prevalence of obesity worldwide, and obesity is associated with excessive storage of lipids (triglycerides) not only in adipose tissue, but also in non-adipose tissues, such as skeletal muscle and liver. Excessive lipid accumulation in the liver is associated with insulin resistance and type 2 diabetes. Lifestyle interventions like exercise training revealed that hepatic fat can be lowered by prolonged exercise training, hence preventing complications associated with hepatic fat accumulation^3^,^4^,^5^,^6^, with patients with high liver fat content showing the greatest reduction following physical activity training. Until now there are no studies investigating the acute effects of exercise on hepatic fat accumulation in an overweight middle-aged population at risk of developing fatty liver or with NAFLD already being present. We show here that an acute bout of exercise did not lead to a detectable decrease IHL, neither in a fasted nor in a glucose-supplemented condition. To our knowledge this is the first study to show this in overweight middle-aged men, which suggests that the long term effects of physical activity training cannot simply be explained by an additive effect of acute exercise bouts.

Whereas IHL did not significantly change within 30 min after cessation of exercise, it was slightly increased 4 h post-exercise in the fasted condition. This finding is consistent with recent reports in young, healthy subjects, in which one exercise session also lead to an increase in IHL after exercise^13^,^14^. The authors suggested that increased FFA availability during exercise^15^,^16^ might be responsible for the increase in IHL. We therefore also examined if exercise would have similar effects on IHL when performed with glucose supplementation during and after exercise. We have previously shown that glucose supplementation during/after exercise markedly blunts the exercise-induced increase in FFA. Consistently, the increase in IHL observed 4 h post exercise was absent in the glucose-supplemented condition. Next to reducing FFA levels, glucose supplementation also increases plasma glucose levels and - although not measured - possibly also insulin levels^20^, and both glucose and insulin are able to stimulate *de novo lipogenesis*^21^, which is an important factor in hepatic fat accumulation^22^. However, since enhanced de novo lipogenesis would result in increased IHL, the absence of an exercise effect on IHL in the glucose fed condition is most likely due to the reduction in plasma FFA levels, suggesting that indeed FFA availability during and after exercise may be an important factor in determining post-exercise IHL content. However, studies directly measuring FFA fluxes into liver are needed to confirm this hypothesis.

Next to measuring the IHL content, ATP/Total P ratio was determined at baseline and 4 h post-exercise in a subgroup of eight subjects. Although, ATP/Total P ratio did not significantly change 4 h post-exercise in neither the fasted nor the glucose-supplemented condition, ATP/Total P ratio had a tendency to be decreased in six out of eight subjects 4 h post-exercise in the fasted condition. It has been suggested that hepatic ATP levels are decreased in conditions such as insulin resistance and type 2 diabetes mellitus^18^,^23^, and that hepatocellular ATP levels are negatively correlating with liver fat in human subjects, suggesting lipotoxicity. In agreement with these data the current study reports a trend to decreased ATP levels along with increased IHL, although no correlation between hepatic lipid content and hepatic ATP levels was found. Interestingly, a recent study investigating the energy charge in the liver of mice after one single bout of exercise found a clear increase of AMP and a strong decrease of ATP in the liver^24^, suggesting that the reduction in ATP levels after exercise is a direct consequence of exercise. In the current study we did observe a non-significant decrease in ATP/Total P ratio after exercise in the fasted condition, whereas no decrease in ATP/Total P ratio was observed after exercise in the glucose-supplemented condition. This suggests that the elevation of IHL upon exercise instead of exercise per se – may be responsible for the reduced hepatic energy status of the liver. However, investigation of larger subject groups will have to determine to what extent the decrease in ATP/Total P ratio is related to increase in IHL.

In summary, we did not observe a decrease in liver fat after acute exercise in overweight middle-aged men. Moreover, IHL was increased post-exercise in the fasted state, an effect that is most likely due to the exercise-induced increase in plasma FFA levels. These data suggest that acute exercise is not responsible for the exercise-lowering effects on liver fat, and that a single bout of exercise may not be able to lower IHL, not even in a population with elevated IHL.

## Methods

### Subjects

Twenty-one middle-aged overweight men with a wide range of liver fat content participated in this study. None of the subjects participated in competitive sports and subjects with unstable body weight (>3 kg change in preceding six months) were excluded from the study. The study protocol conforms to the ethical guidelines of the 1975 Declaration of Helsinki as reflected in a priori approval by the institution's human research committee and written informed consent was obtained from all participants. All methods were carried out in accordance with the approved guidelines and regulations.

### Study protocol

**Figure 5:**
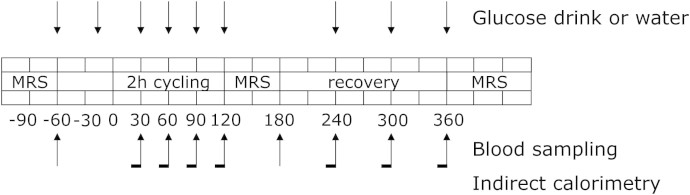
The experimental design of the study. All subjects performed the protocol twice, one time in the fasted state consuming water and one time with glucose supplementation. MRS: hepatic magnetic resonance spectroscopy.

Before the start of the study, body composition and maximal aerobic capacity were determined in all subjects. The experimental trial comprised two separate test days separated by at least one week and performed in random order. Subjects refrained from physical activity two days prior to the test days. Furthermore, subjects were instructed to consume a standardized meal the evening prior to the test days and stayed fasted from 10 pm onwards. On the test days, subjects reported to the laboratory (Maastricht University Medical Center^+^) at 7:00 am after an overnight fast. IHL was investigated by proton magnetic resonance spectroscopy (^1^H-MRS). After this, a Teflon cannula was inserted into an antecubital forearm vein for sampling of blood and subjects rested for 30 minutes. Immediately after drawing the first blood sample, subjects ingested either 1.4 g/kg body weight of glucose (dissolved in water to a 20% solution and flavoured with 1 ml lemon juice) or the same amount of water. After this, subjects started exercising (t = 0 minute) on a stationary bike at 50% of their pre-determined maximal power output (Wmax) for two hours. During exercise, blood samples were drawn and substrate oxidation was measured by indirect calorimetry (Omnical, Maastricht University, Maastricht, The Netherlands) every 30 minutes (at t = 30, 60, 90 and 120 minutes) while heart rate was constantly measured. Immediately after cessation of exercise (t = 120 minute), subjects were transferred to the MR facility for a second ^1^H-MRS measurement. Importantly, due to the transfer time and the preparation steps of the ^1^H-MRS measurement itself, IHL could only be measured within 30 min after cessation of exercise. Subsequently, subjects bed-rested for three hours, followed by a third ^1^H-MRS scan four hours post exercise. Additionally, in a subgroup of eight subjects, ATP/Total P ratio was measured by phosphorous magnetic resonance spectroscopy (^31^P-MRS) at baseline as well as four hours post exercise. During the 4-hour post exercise period, blood samples were drawn and substrate oxidation was measured for 15 minutes every hour (at t = 180, 240, 300 and 360 minutes) in all subjects. The experimental design is depicted in figure 5.

### Measurements prior to test days

A routine incremental cycling test on a stationary bike was used to determine maximal exercise capacity as described previously^25^ and a hydrostatic weighing with simultaneous measurement of lung volume was used to determine body composition. The equation of Siri^26^ was used to calculate fat percentage, fat mass and fat-free mass.

### Blood sample analysis

Blood samples were collected in EDTA-containing tubes and immediately spun at high speed and frozen in liquid nitrogen and subsequently stored at −80°C until assayed. Plasma free fatty acids, triglycerides, and glucose were measured with enzymatic assays automated on a Cobas Fara/Mira (FA: Wako Nefa C test kit; Wako Chemicals, Neuss, Germany) (glucose: hexokinase method; Roche, Basel, Switzerland) (triglycerides: ABX Pentra CP reagents, Horiba ABX Diagnostics, Montpellier, France) (glycerol: Enzytec™ glycerol kit, R-Biopharm, Germany). The liver enzymes Gamma-GT, ASAT and ALAT were routinely measured during the screening visit and analysed in the clinical chemistry department in the hospital.

### ^1^H-MRS

IHL was determined *in vivo* by ^1^H-MRS. Measurements were performed on a whole body scanner (1.5 T, Intera, Philips Healthcare, Best, The Netherlands) as reported previously^27^. An 18 cm^3^ Volume of Interest (VOI) was placed within the lower right hepatic lobe (point resolved spectroscopy (PRESS), repetition time (TR) = 4 s, Echo time (TE) = 23 ms, bandwidth (BW) = 1000 Hz, n = 1024 points, number of signal averages (NSA) = 64). To minimize motion artifacts, subjects were asked to breathe in the rhythm of the measurement and to be at end-expiration during acquisition of spectra. Water signal was suppressed using frequency-selective prepulses. Spectra without water suppression were acquired with identical settings (NSA = 16) and all spectra were fitted with AMARES^28^ in the jMRUI software^29^. The T_2_-corrected ratios of the CH_2_ peak, relative to the unsuppressed T_2_-corrected water resonance was calculated and converted to a tissue fat percentage (weight/weight) by assuming a water content of 71.1%, a CH_2_-proton density of triglycerides of 60.2 mol^−1^, a proton density of water of 2 mol^−1^, a molecular weight of triglycerides and water of 860 g/mol and 18 g/mol, respectively. Three subjects had to be excluded from the IHL analysis due to poor quality of spectra.

### ^31^P-MRS

In a subgroup of eight subjects ATP content was determined at baseline and four hours post exercise in both the fasted and the glucose-supplemented condition. Subjects were positioned in supine position with a 10-cm diameter transmit/receive surface coil positioned at the level of the liver. MRI scout images were acquired during a breath hold and one-dimensional spectroscopic imaging (SI) was performed with 8 phase-encoding steps and SI slice thickness of 30 mm (TR = 4 s, n = 512 points, BW = 4000 Hz, NSA = 16). At least one slice was planned to be exclusively in liver tissue. To minimize motion artifacts, subjects were asked to breathe in the rhythm of the measurement and to be at end-expiration during acquisition of spectra. All spectra were fitted with the AMARES algorithm^28^ in the jMRUI software package^29^. The γ-ATP resonance was quantified and expressed as ratio of the γ-ATP to total phosphorus signal in the −25 to 25 ppm frequency region, expressed as ATP/Total P ratio in the current paper.

### Statistics

Data are presented as mean ± SE. Hepatic lipid content was not normally distributed and therefore a non-parametric Friedman test was performed to test if there was an overall effect on IHL. Thereafter a pairwise comparison was performed with a Bonferroni correction for multiple comparisons. A two-way repeated measures ANOVA was performed to compare the mean differences between conditions for ATP, substrate oxidation and plasma values of FA, triglycerides, and glucose. All statistics were performed using SPSS 16.0 (IBM Corporation, Armonk, NY, USA) for Mac and p < 0.05 was considered statistically significant.

## Author Contributions

L.B. and B.B. have set up the study concept and design, performed acquisition, analysis and interpretation of data, were responsible for the draft of the manuscript and performed statistical analysis; P.A.E. has set up the ^31^P-MRS protocol and performed acquisition, analysis and interpretation of data; M.K.C.H. has set up the study concept and design and was responsible for the draft of the manuscript and supervision of the study process; M.E.K. has set up the ^31^P-MRS protocol and performed analysis and interpretation of data; P.S. and V.B.S.H. have set up the study concept and design, interpreted the data, were responsible for the draft of the manuscript and supervision of the study process and obtained funding. All authors reviewed the manuscript.

## Figures and Tables

**Table 1:** Subject characteristics

Mean ± SD	Liver fat < 5.6%	Liver fat > 5.6%	All subjects (pooled)
Age, yr	58.2 ± 7.7	51.7 ± 5.4*	54.8 ± 7.2
Height, m	1.79 ± 0.05	1.79 ± 0.03	1.79 ± 0.04
Weight, kg	92.6 ± 9.1	97.5 ± 8.6	95.2 ± 9.0
BMI, kg/m^2^	28.7 ± 1.8	30.4 ± 2.3	29.6 ± 2.2
Fat %	27.5 ± 6.2	28.5 ± 3.1	27.9 ± 4.9
VO_2max_/kg,ml × min^−1^ × kg^1^	30.3 ± 5.7	29.1 ± 6.1	29.7 ± 5.8
SBP (mmHg)	135.7 ± 16.5	144.7 ± 12.6	140.4 ± 15.0
DBP (mmHg)	83.3 ± 12.0	95.1 ± 6.7*	89.5 ± 11.1
Glucose (mmol/L)	5.3 ± 0.8	5.6 ± 0.4	5.4 ± 0.6
Gamma-GT (U/L)	27.6 ± 9.4	55.5 ± 26.2*	42.2 ± 24.2
ASAT (U/L)	18.0 ± 4.5	25.9 ± 6.6*	22.1 ± 6.9
ALAT (U/L)	25.4 ± 8.8	44.6 ± 11.0*	35.5 ± 13.9
Triglycerides (mmol/L)	1.1 ± 0.3	2.0 ± 0.5*	1.5 ± 0.6
Liver fat (%)	2.7 ± 1.5	13.1 ± 6.7*	8.1 ± 7.2

Data are means ± SD. BMI, Body Mass Index; SBP, Systolic Blood Pressure; DBP, Diastolic Blood Pressure. * p < 0.05 compared with subjects having less than 5.6% liver fat.

**Table 2:** Energy expenditure and fat and carbohydrate oxidation during and after exercise in glucose-supplemented and fasted condition

		Glucose-supplemented			Fasted		
	Time, min	EE, kJ/min	Fat oxidation, mg/min	CHO oxidation, mg/min	EE, kJ/min	Fat oxidation, mg/min	CHO oxidation, mg/min
Exercise	30	36 ± 4	342 ± 108#	1442 ± 273#	36 ± 5	457 ± 139	1125 ± 337
	60	37 ± 4	512 ± 133#	1077 ± 362#	37 ± 5	592 ± 162	854 ± 396
	90	36 ± 5	534 ± 114#	942 ± 326#	36 ± 5	646 ± 174	666 ± 424
	120	35 ± 4	548 ± 135#	891 ± 329#	36 ± 6	672 ± 164	600 ± 438
Post-exercise	240	5 ± 1	54 ± 43#	231 ± 107#	6 ± 1	107 ± 21	79 ± 45
	300	5 ± 1	45 ± 20#	238 ± 54#	5 ± 1	108 ± 22	72 ± 45
	360	5 ± 1	45 ± 18#	240 ± 45#	6 ± 1	114 ± 21	70 ± 45

Data are means ± SE, n = 17. EE, energy expenditure; CHO, carbohydrate. # p < 0.01 compared with fasted condition.

## References

[b1] Dela F. Other adaptations to training/inactivity in type 2 diabetics and other groups with insulin resistance: emphasis on prevention of CHD. Appl Physiol Nutr Metab 32, 602–606 (2007). 17510702 10.1139/H07-028

[b2] Gordon B. A., Benson A. C., Bird S. R. & Fraser S. F. Resistance training improves metabolic health in type 2 diabetes: a systematic review. Diabetes Res Clin Pract 83, 157–175 (2009). 19135754 10.1016/j.diabres.2008.11.024

[b3] Sullivan S., Kirk E. P., Mittendorfer B., Patterson B. W. & Klein S. Randomized trial of exercise effect on intrahepatic triglyceride content and lipid kinetics in nonalcoholic fatty liver disease. Hepatology 55, 1738–1745 (2012). 22213436 10.1002/hep.25548 PMC3337888

[b4] Hallsworth K. *et al.* Resistance exercise reduces liver fat and its mediators in non-alcoholic fatty liver disease independent of weight loss. Gut 60, 1278–1283 (2011). 21708823 10.1136/gut.2011.242073 PMC3152868

[b5] Finucane F. M. *et al.* The effects of aerobic exercise on metabolic risk, insulin sensitivity and intrahepatic lipid in healthy older people from the Hertfordshire Cohort Study: a randomised controlled trial. Diabetologia 53, 624–631 (2010). 20052455 10.1007/s00125-009-1641-z

[b6] Chen S. M. *et al.* Effects of therapeutic lifestyle program on ultrasound-diagnosed nonalcoholic fatty liver disease. J Chin Med Assoc 71, 551–558 (2008). 19015052 10.1016/S1726-4901(08)70168-0

[b7] Magkos F. Putative factors that may modulate the effect of exercise on liver fat: insights from animal studies. J Nutr Metab 2012, 827417, 10.1155/2012/827417 (2012). 21912741 PMC3168901

[b8] Magkos F. Exercise and fat accumulation in the human liver. Curr Opin Lipidol 21, 507–517, 10.1097/MOL.0b013e32833ea912 (2010). 21206340

[b9] Rogers M. A. Acute effects of exercise on glucose tolerance in non-insulin-dependent diabetes. Med Sci Sports Exerc 21, 362–368 (1989). 2674587

[b10] Frank P., Katz A., Andersson E. & Sahlin K. Acute exercise reverses starvation-mediated insulin resistance in humans. Am J Physiol Endocrinol Metab 304, E436–443, 10.1152/ajpendo.00416.2012 (2013). 23269410

[b11] Mackenzie R. *et al.* Intermittent exercise with and without hypoxia improves insulin sensitivity in individuals with type 2 diabetes. J Clin Endocrinol Metab 97, E546–555, 10.1210/jc.2011-2829 (2012). 22278428

[b12] Johnson N. A. *et al.* Effect of prolonged exercise and pre-exercise dietary manipulation on hepatic triglycerides in trained men. Eur J Appl Physiol 112, 1817–1825 (2011). 21915700 10.1007/s00421-011-2158-y

[b13] Bucher J. *et al.* The effect of a single 2 h bout of aerobic exercise on ectopic lipids in skeletal muscle, liver and the myocardium. Diabetologia, 10.1007/s00125-014-3193-0 (2014). 24563325

[b14] Egger A. *et al.* The effect of aerobic exercise on intrahepatocellular and intramyocellular lipids in healthy subjects. PLoS One 8, e70865, 10.1371/journal.pone.0070865 (2013). 23967125 PMC3743875

[b15] Schrauwen P. *et al.* Effect of acute exercise on uncoupling protein 3 is a fat metabolism-mediated effect. Am J Physiol Endocrinol Metab 282, E11–17 (2002). 11739077 10.1152/ajpendo.2002.282.1.E11

[b16] Bilet L. *et al.* Exercise-induced modulation of cardiac lipid content in healthy lean young men. Basic Res Cardiol 106, 307–315 (2010). 21181177 10.1007/s00395-010-0144-x PMC3032894

[b17] Donnelly K. L. *et al.* Sources of fatty acids stored in liver and secreted via lipoproteins in patients with nonalcoholic fatty liver disease. J Clin Invest 115, 1343–1351 (2005). 15864352 10.1172/JCI23621 PMC1087172

[b18] Szendroedi J. *et al.* Abnormal hepatic energy homeostasis in type 2 diabetes. Hepatology 50, 1079–1086 (2009). 19637187 10.1002/hep.23093

[b19] Szczepaniak L. S. *et al.* Magnetic resonance spectroscopy to measure hepatic triglyceride content: prevalence of hepatic steatosis in the general population. Am J Physiol Endocrinol Metab 288, E462–468 (2005). 15339742 10.1152/ajpendo.00064.2004

[b20] Stellingwerff T. *et al.* Carbohydrate supplementation during prolonged cycling exercise spares muscle glycogen but does not affect intramyocellular lipid use. Pflugers Arch 454, 635–647, 10.1007/s00424-007-0236-0 (2007). 17333244 PMC1915642

[b21] Hellerstein M. K. De novo lipogenesis in humans: metabolic and regulatory aspects. Eur J Clin Nutr 53 Suppl 1, S53–65 (1999). 10365981 10.1038/sj.ejcn.1600744

[b22] Ferre P. & Foufelle F. Hepatic steatosis: a role for de novo lipogenesis and the transcription factor SREBP-1c. Diabetes Obes Metab 12 Suppl 2, 83–92, 10.1111/j.1463-1326.2010.01275.x (2010). 21029304

[b23] Schmid A. I. *et al.* Liver ATP synthesis is lower and relates to insulin sensitivity in patients with type 2 diabetes. Diabetes Care 34, 448–453 (2011). 21216854 10.2337/dc10-1076 PMC3024365

[b24] Camacho R. C., Donahue E. P., James F. D., Berglund E. D. & Wasserman D. H. Energy state of the liver during short-term and exhaustive exercise in C57BL/6J mice. Am J Physiol Endocrinol Metab 290, E405–408, 10.1152/ajpendo.00385.2005 (2006). 16219665

[b25] Kuipers H., Verstappen F. T., Keizer H. A., Geurten P. & van Kranenburg G. Variability of aerobic performance in the laboratory and its physiologic correlates. Int J Sports Med 6, 197–201, 10.1055/s-2008-1025839 (1985). 4044103

[b26] Siri W. E. The gross composition of the body. Adv Biol Med Phys 4, 239–280 (1956). 13354513 10.1016/b978-1-4832-3110-5.50011-x

[b27] van Herpen N. A., Schrauwen-Hinderling V. B., Schaart G., Mensink R. P. & Schrauwen P. Three weeks on a high-fat diet increases intrahepatic lipid accumulation and decreases metabolic flexibility in healthy overweight men. J Clin Endocrinol Metab 96, E691–695, 10.1210/jc.2010-2243 (2011). 21252252

[b28] Vanhamme L., van den Boogaart A. & Van Huffel S. Improved method for accurate and efficient quantification of MRS data with use of prior knowledge. J Magn Reson 129, 35–43 (1997). 9405214 10.1006/jmre.1997.1244

[b29] Naressi A. *et al.* Java-based graphical user interface for the MRUI quantitation package. Magma 12, 141–152 (2001). 11390270 10.1007/BF02668096

